# MHC-II presentation by oral Langerhans cells impacts intraepithelial Tc17 abundance and *Candida albicans* oral infection via CD4 T cells

**DOI:** 10.3389/froh.2024.1408255

**Published:** 2024-05-30

**Authors:** Peter D. Bittner-Eddy, Lori A. Fischer, Praveen Venkata Parachuru, Massimo Costalonga

**Affiliations:** ^1^ Division of Basic Sciences, Department of Diagnostic and Biological Sciences, School of Dentistry, University of Minnesota, Minneapolis, MN, United States; ^2^ Division of Periodontology, Department of Developmental and Surgical Sciences, School of Dentistry, University of Minnesota, Minneapolis, MN, United States

**Keywords:** Langerhans cells, *Candida albicans*, T cytotoxic 17 cells (Tc17), CD4 T cells, MHC-II antigen presentation, oral mucosa

## Abstract

In a murine model (LC^ΔMHC-II^) designed to abolish MHC-II expression in Langerhans cells (LCs), ∼18% of oral LCs retain MHC-II, yet oral mucosal CD4 T cells numbers are unaffected. In LC^ΔMHC-II^ mice, we now show that oral intraepithelial conventional CD8αβ T cell numbers expand 30-fold. Antibody-mediated ablation of CD4 T cells in wild-type mice also resulted in CD8αβ T cell expansion in the oral mucosa. Therefore, we *hypothesize* that MHC class II molecules uniquely expressed on Langerhans cells mediate the suppression of intraepithelial resident-memory CD8 T cell numbers via a CD4 T cell-dependent mechanism. The expanded oral CD8 T cells co-expressed CD69 and CD103 and the majority produced IL-17A [CD8 T cytotoxic (Tc)17 cells] with a minority expressing IFN-γ (Tc1 cells). These oral CD8 T cells showed broad T cell receptor Vβ gene usage indicating responsiveness to diverse oral antigens. Generally supporting Tc17 cells, transforming growth factor-β1 (TGF-β1) increased 4-fold in the oral mucosa. Surprisingly, blocking TGF-β1 signaling with the TGF-R1 kinase inhibitor, LY364947, did not reduce Tc17 or Tc1 numbers. Nonetheless, LY364947 increased γδ T cell numbers and decreased CD49a expression on Tc1 cells. Although IL-17A-expressing γδ T cells were reduced by 30%, LC^ΔMHC-II^ mice displayed greater resistance to *Candida albicans* in early stages of oral infection. These findings suggest that modulating MHC-II expression in oral LC may be an effective strategy against fungal infections at mucosal surfaces counteracted by IL-17A-dependent mechanisms.

## Introduction

The gut, skin, and oral cavity are major physical and immunological barriers to infection by pathogens and sites of immune encounters with beneficial microbiota. These barrier tissues have distinct assemblages of immune cells each adapted to respond in an appropriate manner to site-specific challenges. The stratified squamous epithelia of the skin and oral mucosa contain a unique population of innate immune cells called Langerhans cells (LCs), which capture antigens on major histocompatibility complex class II molecules (MHC-II) and then present to antigen-experienced CD4 T cells locally and to naïve CD4 T cells in draining lymph nodes (1, 2). LCs also direct CD4 T helper 17 (Th17) responses against extracellular pathogens (3, 4) and, potentially regulate specific immunity (5, 6). Although skin-resident and oral LCs occupy a similar niche in stratified epithelial barriers, have similar transcriptomic profiles and immunologic functions, they have distinctive ontogenetic differences (2, 7). Skin-resident LCs are involved in many skin disorders and contribute actively to defense against cutaneous infections with *Candida albicans* (1, 3).

Our understanding of the acute immune response to *C. albicans* comes mainly from murine studies (8, 9). Mice lacking T cell receptors appear highly susceptible to *C. albicans* infection implicating CD4 and/or CD8 T cells in facilitating resistance (8). In humans, *C. albicans-*specific memory T cells are largely Th17, and in mice IL-17A is critical for *C. albicans* clearance (9–11). IL-17A stimulates epithelial cells to produce antimicrobial peptides (12) and the neutrophil-recruiting chemokines C-X-C Motif Chemokine Ligand 8 (CXCL8) and granulocyte-monocyte colony stimulating factor (GM-CSF) (13). In mouse models of oral candidiasis, Th17 cells control initiation of local infection while Th1 cells prevent systemic spread (11, 14, 15). In addition to Th17 cells, innate cells of lymphoid origin can produce IL-17A, including γδ T, natural killer T, innate lymphoid cell type 3, TCR-β^+^ “natural” Th17 cells, and a population of major histocompatibility complex class I (MHC-I)-restricted CD8 T cells (Tc17) (8, 16). Tc17 cells produce reduced amounts of prototypical IFN-γ and cytotoxic molecules (17) and function as a potential novel source of IL-17A in the presence of low numbers of Th17 cells.

The ontogeny of Tc17 cells *in vivo* is not yet fully understood, but *in vitro* work suggests the critical importance of TGF-β and IL-6 in the differentiation of naïve CD8 T cells into Tc17 cells (17). For example, allochthonous microorganisms entering the cutaneous microbial flora increase Tc17 cell abundance, conferring IL-17A-dependent protection against *C. albicans* in a skin infection model (18). Moreover, in a CD4^−/−^ mouse model that mimics the depressed CD4 T cell status of HIV-positive individuals, Tc17 cells were numerous and helped confer resistance to *C. albicans* in an oropharyngeal candidiasis infection model (19). In the oral epithelial lining, therefore, Tc17 cells appear to deliver potent anti-fungal immunity by providing compensatory IL-17A in immunocompromised CD4 T cell-deficient individuals. Interestingly, cultured Tc17 cells have a central memory phenotype and become plastic, shifting into the prototypical cytotoxic type 1 CD8 T cells (Tc1) when adoptively transferred and subsequently challenged *in vivo* (20).

In both humans and mice, oral dendritic cell subsets in direct innate and memory immune responses against *C. albicans* (16). These cell populations may contribute to defense against opportunistic oropharyngeal candidiasis affecting the oral mucosa, tongue, and the lining of the esophagus*.* In the development of a protective mucosal Tc17 population against *C. albicans* infection, the roles of mucosal antigen presenting cells including conventional dendritic cells, subepithelial langerin^+^ dendritic cells, and oral intraepithelial LCs have yet to be defined. LCs drive antigen-specific Th17 responses against *C. albicans* in the skin and against a periodontal pathogen in the oral cavity (3, 4). We hypothesized that MHC class II molecules uniquely expressed on LCs mediate the suppression of intraepithelial resident-memory CD8 T cell numbers that primarily express IL-17A to affect the susceptibility to *C. albicans* infection. Functional redundancy seems to occur in oral LC antigen presentation. An 80% reduction in cells expressing MHC-II has no impact on the development of an oral Th17 response in either specific-pathogen free conditions or in a ligature-induced periodontitis model (21). Interestingly, reduced numbers of MHC-II-expressing oral LCs increased oral CD8 T cells by a striking 30-fold (21). To better understand their function, we further characterize these CD8 T cells to be IL-17A-expressing Tc17 cells with a resident-memory phenotype localized within the suprabasal layer of the oral mucosa epithelium. This dramatic increase in oral Tc17 numbers regulated by MHC class II expression on LCs translated into increased protection against oropharyngeal candidiasis induced by *C. albicans* infection.

## Materials and methods

### Mice

Mice were housed in AAALAC approved specific-pathogen free conditions at the Animal Facilities of the University of Minnesota and their use approved by the Institutional Animal Care and Use Committee (Protocol 2110-39519A). All mice used are on the C57BL/6J genetic background. Generation of LC^WT^, LC^ΔMHC-II^ and IL-17A^Cre-RFP^ reporter mice have been described elsewhere (21, 22). A heterozygous breeding strategy was used to produce sibling experimental LC^WT^ and LC^ΔMHC-II^ animals and genotypes were confirmed using PCR to detect segregation of the huLang-Cre transgene amongst littermates (21). Experiments were performed on age-matched (8–12 week) mice of both sexes unless specifically stated otherwise. Mice were co-housed in microisolator cages with a 12-h day/night cycle, standard chow, and water *ad libitum*.

### Preparation of single cell suspensions from mouse tissues and flow cytometry

Mice were routinely injected retroorbitally with 1.25 µg of rat anti-mouse CD45 mAb conjugated to FITC (BioLegend; clone 30-F11) 3 min prior to euthanasia to discriminate blood-resident immune cells from interstitial immune cells as previously described (23).

Oral mucosa was harvested as previously described, judiciously avoiding potential nasal associated lymphoid tissue contamination (23). In some experiments, oral mucosa was subsequently treated with Dispase II to separate the epithelium from the underlying lamina propria and tissues processed separately (21). Briefly, oral mucosa was incubated in 2.2 U/ml Dispase II (Roche) at 37 °C with gentle agitation for 50 min. Fine tipped jewelry tweezers were then used to peel away epithelial sheets from the lamina propria. Minced oral mucosa/lamina propria and epithelial sheets were further treated with collagenase D (Roche) and DNase I (Sigma-Aldrich) before processing tissues to obtain single cell suspensions for flow cytometry as previously described (23).

A scalpel was used to manually remove and separate the dermis and epidermis from both the dorsal and ventral surfaces of the left ear for skin samples. Excised lungs were first rinsed in ice cold PBS. Skin and lung specimens were finely minced and then digested with collagenase D (2 mg/ml) and DNase I (1 mg/ml) at 37 °C for 60 min before processing to obtain single cell suspensions using standard procedures.

Cervical lymph nodes and spleen were harvested and processed to produce single cell suspensions using standard techniques as previously described (23). Single cell suspensions in all flow cytometry experiments were first stained with viability dye Zombie Aqua (BioLegend) followed by incubation with rat anti-mouse CD16/CD32 mAb (eBioscience; clone 93) to block Fc receptors. Cells were then incubated with combinations of the following rat/hamster anti-mouse fluorochrome-conjugated mAbs (BioLegend): CD45 (clone 30-F11), CD3 (clone 17A2), CD4 (clone RM4-5), CD8α (clone 53-6.7), CD8β (clone YTS156.7.7), CD90.2 (clone 30-H11), TCRβ (clone H57-597), TCRγ/δ (clone GL3), CD25 (eBioscience; clone PC61), CD44 (clone IM7), CD69 (clone H1.2F3), CD103 (clone 2E7), CD49a (clone HMa1). In experiments to identify potential mucosal-associated invariant T (MAIT) cells, single cell suspensions were additionally stained with major histocompatibility complex (MHC) class I-related protein 1 (MR1) tetramers, MR1 5-(2-oxopropylideneamino)-6-D-ribitylaminouracil (5-OP-RU) or MR1 6-formyl pterin (6-FP) purchased from the NIH tetramer facility based at Emory University, Atlanta, Georgia. Staining was performed in accordance with NIH tetramer facility instructions. In flow cytometry experiments using mucosal tissue, cells were pre-filtered through 70 µm cell strainers to remove cell clumps/debris. All cells were acquired on an LSR II flow cytometer (BD Biosciences) equipped with 4 lasers. Fluorescence emissions were analyzed using FlowJo 10.7.1 (Tree Star). To account for sample loss during mAb staining and/or variability in processed tissue size, immune cell populations of interest were normalized to 100,000 live non-immune cells when appropriate.

### Depletion of CD4 T cells

Eight-10 week old IL-17A^Cre-RFP^ reporter mice were injected i.p. with 200 µg anti-CD4 mAb (BioXCell; clone GK1.5) or Rat IgG2b *κ* control (BioXCell; #BE0090) 4 times, 5 days apart. On day 20, mice were injected with FITC-labeled anti-CD45 mAb, sacrificed 3 min later, and oral mucosal tissue processed to generate single cell suspensions as described above. As a tissue lacking LCs (control tissue), the left lung was perfused with PBS, minced, treated with collagenase D and DNAse I and the resulting single cell suspensions further treated with ACK (Lonza) to lyse red blood cells. All single cell suspensions were subsequently stained with mAbs to identify CD4 T cells and CD8 T cells by flow cytometry as described above.

### T cell phenotyping and TCR Vβ usage

To determine the phenotype of T cells, single cell suspensions were cultured in complete EHAA (Irvine Scientific) at 37 °C and polyclonally stimulated for 6 h with PMA-Ionomycin in the presence of brefeldin A as previously described (23). Cells were then incubated with Zombie Aqua, anti-mouse CD16/CD32 antibody and surface stained with mAbs as described above. Cells were fixed and made permeable using BD Cytoperm/Cytofix Plus kit reagents according to the manufacturer's instructions (BD Biosciences). Permeabilized cells were subsequently stained with anti-mouse IL-17A (eBioscience; clone eBio17B7) and IFN-γ (BioLegend; clone XMG1.2) fluorochrome-conjugated mAbs.

TCR Vβ gene usage was determined using a mouse Vβ TCR screening panel kit that targets the use of 17 different TCR Vβ alleles (BD Biosciences). Oral mucosa from 12 LC^ΔMHC-II^ mice was harvested, treated with Dispase II to separate the epithelium from the underlying lamina propria and single cell suspensions produced from pooled epithelial sheets. Cells were stained with mAbs to specifically identify CD4 T and CD8 T cells, then washed and stained with FITC-conjugated mAbs that recognize the 16 different TCRs in C57BL/6 mice following the manufacturer's instructions. In some experiments, cells were additionally stained with rat anti-mouse CD49a mAb prior to use of the Vβ TCR panel. Since the *V*β *17a* gene is not expressed in the C57BL/6J mouse, the anti-Vβ 17a mAb can be viewed as a negative staining control.

### Preparation of oral mucosa and epithelial sheets from tongue for confocal microscopy

Oral mucosal tissue was harvested as previously described (23), subsequently embedded in O.C.T. medium (Sakura Finetek) and 10 µm frozen cryostat sections cut and fixed with 4% paraformaldehyde. Sections were briefly re-hydrated in PBS and then blocked with PBS supplemented with 5% (v/v) rat serum and 5% (v/v) FBS for 60 min at room temperature and then incubated overnight at 4°C with 10 µg/ml Alexa Fluor 647 anti-mouse CD8α mAb (BioLegend; clone 53-6.7) in PBS 5% (v/v) rat serum. After two washes with PBS 0.1% (v/v) Tween 20, sections were counterstained with DAPI (Life Technologies) and imaged at 10–40× using a LSM 700 Zeiss confocal laser scanning microscope (Carl Zeiss).

Tongues were excised and much of the underlying musculature removed and discarded. Processed tongues were then treated with 2.2 U/ml Dispase II (Roche) for 50 min at 37 °C to allow the epithelium to be detached as a sheet from the remaining muscle. Epithelial sheets were fixed with 4% paraformaldehyde, re-hydrated in PBS, and then blocked with PBS supplemented with 5% (v/v) rat serum, 5% (v/v) FBS, and 0.1% (v/v) Tween 20 for 60 min at room temperature. Epithelial sheets were then incubated overnight at 4 °C with 10 µg/ml Alexa Fluor 647 anti-mouse/human CD207 mAb (Dendritics; clone 929F3.01) and 10 µg/ml Spark YG 570 anti-mouse CD8α mAb (BioLegend; clone 53-6.7) in PBS 5% (v/v) rat serum, and 0.1% (v/v) Tween 20. After two washes with PBS 0.1% (v/v) Tween 20, sections were counterstained with DAPI (Life Technologies), mounted in 100% glycerol and imaged at 10–40× using a LSM 700 Zeiss confocal laser scanning microscope (Carl Zeiss). Fiji software (Image J 2.1.0) was used to create single 2D images from captured *Z* stacks. Adjoining images were manually stitched together using Adobe Photoshop (Adobe Inc).

### qPCR analysis of oral mucosa tissue

Total RNA was extracted from oral mucosa using a Master Pure Complete DNA and RNA Purification kit (Epicentre Technologies) and contaminating DNA removed following treatment of samples with AmbionTM Turbo DNase (ThermoFischer Scientific). RNA was reverse transcribed into cDNA using the ProtoScript II First Strand cDNA Synthesis Kit (New England BioLabs Inc.) according to the manufacturer's instructions.

cDNA samples were analyzed in triplicate for each gene of interest using a MX3000P Stratagene Real Time qPCR instrument (Agilent Technologies). Each qPCR reaction (final volume, 25 μl) contained 12.5 μl of SYBR Green Supermix (Bio-Rad Laboratories), 1 ml of each primer (0.3 μm), 5.5 μl of Nuclease-free water and 20 ng/5 μl of cDNA. A zero-template control was included in each assay. The thermo-cycling program consisted of one hold at 95 °C for 10 min followed by 40 cycles of 15 s at 95 °C, 30 s at 60 °C, 30 s at 72 °C. A melting curve protocol was followed to determine the specificity of the product and to rule out primer-dimer formation or potential amplification of contaminating DNA. All qPCR primers used flanked introns. Primer sequence, binding position within cDNA, intron flanked and expected amplicon size are given in Supplementary Table S1.

From a panel of 5 potential housekeeping genes, hydroxymethylbilane synthase was determined to be the most stable in our samples as described (24). For the purposes of our analysis, a Cq value ≥35 was treated as gene expression not detected. The ΔΔCq method (25) was used to analyze the raw Cq values following normalization to expression of the housekeeping gene, hydroxymethylbilane synthase. Fold change (2-^ΔΔCq^) in gene expression was determined by comparison of the mean of the normalized expression values, between LC^ΔMHC-II^ and control LC^WT^ groups, using the ΔΔCq method (25). Unpaired two-tailed Student's *t*-test was used to analyze the difference in the expression level of the tested genes between the groups. Genes with a statistical significance of ±2-fold change and a *p* value <0.05 were considered as differentially regulated.

### Inhibition of TGFβ signaling using LY364947

The inhibitor of TGFβ receptor signaling (26) LY364947 (Selleckchem) was dissolved in DMSO to a concentration of 20 mg/ml, and aliquots stored at −80 °C. Working LY364947 solutions (0.4 mg/ml in PBS 2% DMSO) were prepared fresh from −80 °C stocks on the day on injection. LY364947 was injected intraperitoneally once a day for 7 days at a dose of 4 mg per kg body weight and mice were euthanized 24 h following the final LY364947 injection. Control mice received similar volumes of PBS 2% DMSO vehicle over the same injection regimen. Oral mucosa was harvested, and single cell suspensions produced from epithelial sheets for analysis by flow cytometry.

### Oral infection of mice with *Candida albicans* and bioluminescence imaging

Mice were orally infected with *C. albicans* using an established co-infection model without immunosuppression (27). *Streptococcus oralis* 34, a gift from Anna Dongari-Bagtzoglou (University of Connecticut), was maintained on BHI broth agar plates (Research Products International). *C. albicans* strain DSY4976 stably expressing a red-shifted firefly luciferase gene and the non-transformed parental strain DSY49709 (28) were a gift from Dominique Sanglard (University of Lausanne) and were maintained on Yeast Peptone Dextrose (YPD) (1% yeast extract Fluka Analytical; 2% Bacto Peptone BD Biosciences; 2% Dextrose Sigma Aldrich) agar plates (Research Products International). Single colonies of *C. albicans* and *S. oralis* were utilized to establish liquid cultures for inoculum production. *S. oralis* was grown o/n in brain-heart infusion (BHI) broth (37 °C, 5% CO_2_, static) and cfu determined by optical density at 600 nm (OD600 nm reading of 1 = 1 × 10^8^ cfu/ml). *C. albicans* was grown o/n in YPD broth in a shaking incubator at 28 °C to maintain growth as yeast cells. *C. albicans* and *S. oralis* were collected by centrifugation (537 g for 10 min at room temperature) and the cell pellets washed twice with room temperature Hank's Balanced Salt Solution (HBSS) (Gibco Life Technologies). After the final wash, *S. oralis* pellets were resuspended in HBSS at a concentration of 10 × 10^9^ cfu/ml. Final *C. albicans* pellets were resuspended in a small volume of HBSS, spores counted, and the concentration adjusted to 2.4 × 10^9^ cfu/ml in HBSS. The inoculum was prepared by mixing an equal volume of *C. albicans* and *S. oralis* in a 1.5 ml tube and placing the tube in a 30 °C heat block until inoculation.

Mice were anesthetized with a ketamine/xylazine mixture and placed in a supine position. Fifty µl of inoculum was applied to the applicator tip of a calcium alginate swab (Puritan) and the swab placed in the oral cavity on top of the tongue. Swabs were removed at 60 min. When required, bioluminescence was captured in live mice using an *in vivo* imaging system (IVIS) Spectrum (PerkinElmer). Briefly, 10 µl of D-Luciferin (PerkinElmer) substrate at 1.5 mg/ml was placed into the oral cavity of isoflurane-anesthetized mice and bioluminescence signal captured at 5 min using the following IVIS Spectrum settings: FOV = D; F1; Bin = 4; 5-min exposure. Images were analyzed using Living Image software (PerkinElmer). Region of interest (ROI) was established as 30 × 30 pixels (1.95 cm^2^) and a minimum threshold of 600 counts was set for all experiments. Radiance efficiency was captured for each ROI and expressed as total flux (photons/s). Background bioluminescence (∼3 × 10^4^ total flux photons/s) was established in mice orally infected with the non-luciferase parental *C. albicans* strain DSY49709.

### Statistical analysis

Data was analyzed and plotted using Prism 8 software (GraphPad Software) and displayed as means ± SEM or individual data points in plots showing correlations. Significance between two groups was determined by unpaired two-tailed Student's *t*-test or Mann–Whitney *U*-test when data did not show Gaussian distribution (Shapiro–Wilk normality test). Correlations were determined from computed Pearson correlation coefficients. *p* values less than 0.05 were considered significant. Biological sex was not considered a significant variable except in our oropharyngeal candidiasis infection model.

## Results

### CD8 T cells are located predominately in the suprabasal layer of the oral mucosa epithelium in LC^ΔMHC-II^ mice

**Figure 1:**
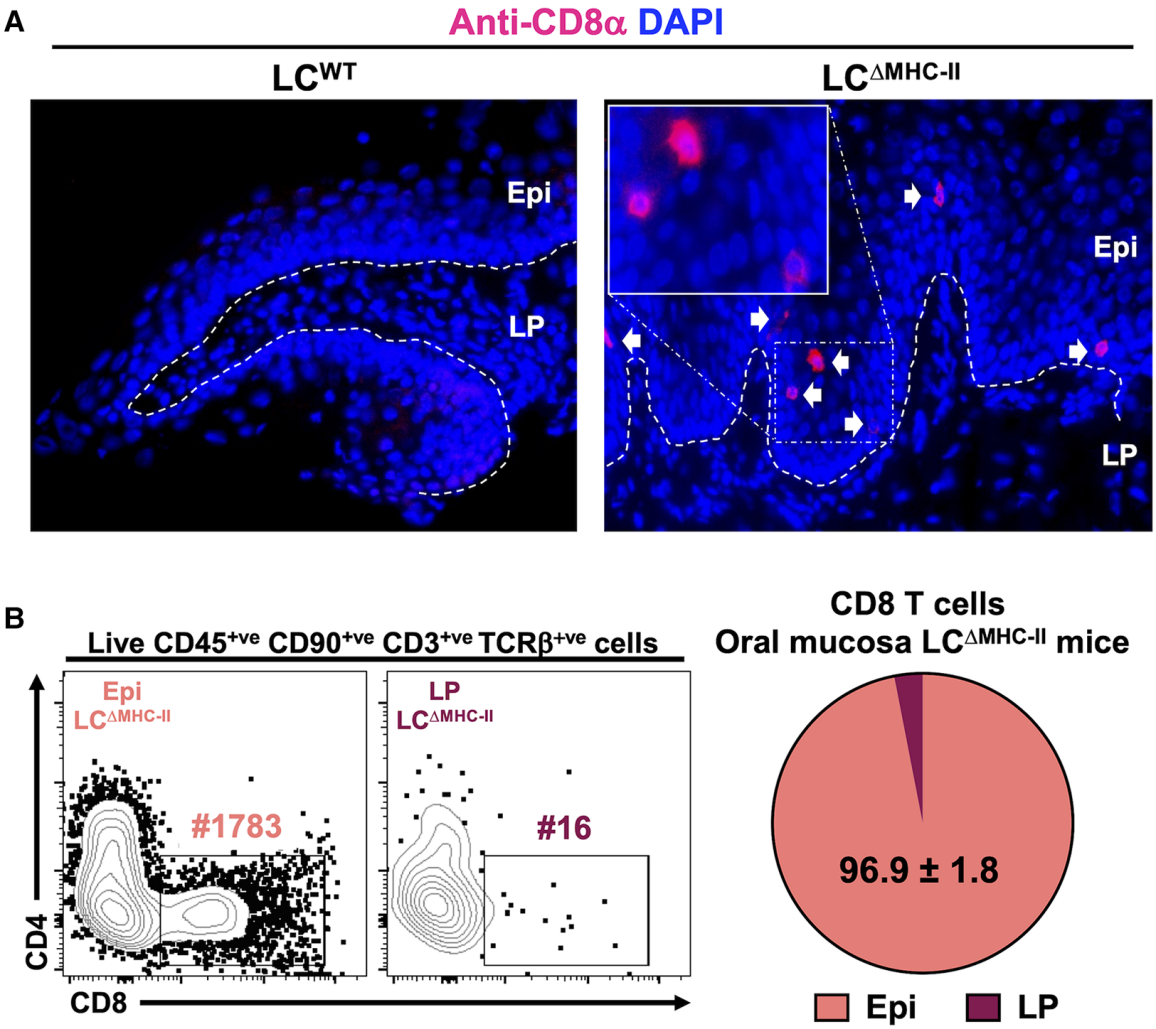
CD8 T cells in the oral mucosa of LC^ΔMHC-II^ mice reside predominantly within the suprabasal epithelial layer. (**A**) Ten µm oral mucosa frozen sections were stained with DAPI and rat anti-mouse CD8α mAb conjugated to Alexa Fluor 647. Representative images obtained from a LC^ΔMHC-II^ mouse and a huLang Cre negative “WT” littermate control (LC^WT^) are shown. White arrows indicate CD8 T cells located within the oral mucosal epithelium. Segmented white line separates Epi (epithelium) from LP (lamina propria). Scale bar is 50 µm. (**B**) Oral mucosa from LC^ΔMHC-II^ mice were treated with Dispase II to separate the tissue specimen into epithelium and underlining lamina propria components. Single cell suspensions were obtained from the two tissue fractions and stained with rat anti-mouse mAbs for flow cytometry. Live CD8 T cells were identified as Zombie Aqua^lo^, CD45^+^, CD90.2^+^, CD3^+^, TCRβ^+^, CD8α^+^ and CD4^−^. Representative flow cytometry plots from Epi and LP samples are shown including the number of CD8 T cells (#) for each sample. Summary data from 4 LC^ΔMHC-II^ mice are displayed showing the mean percentage ± SEM of CD8 T cells found in the Epi or LP.

Using flow cytometry, we showed CD8 T cells to be significantly increased in the oral mucosa of mice (LC^ΔMHC-II^) engineered to ablate MHC-II on LCs (21). In LC^ΔMHC-II^ mice the enhanced number of CD8α^+^ cells were located within the stratified mucosal epithelium and almost exclusively within the suprabasal layer (Figure 1A). To quantify CD8 T cell distribution in the mucosal tissue, we separated the stratified epithelium from the underlying lamina propria. Virtually all CD8 T cells (96.9%) from LC^ΔMHC-II^ mice are located within the stratified mucosal epithelium (Figure 1B; Supplementary Figure S1), consistent with our immunohistology observations. In LC^WT^ mice there were insufficient CD8 T cells to perform a similar analysis.

### CD8 T cell numbers are increased in the skin of LC^ΔMHC-II^ mice but not in lung

**Figure 2:**
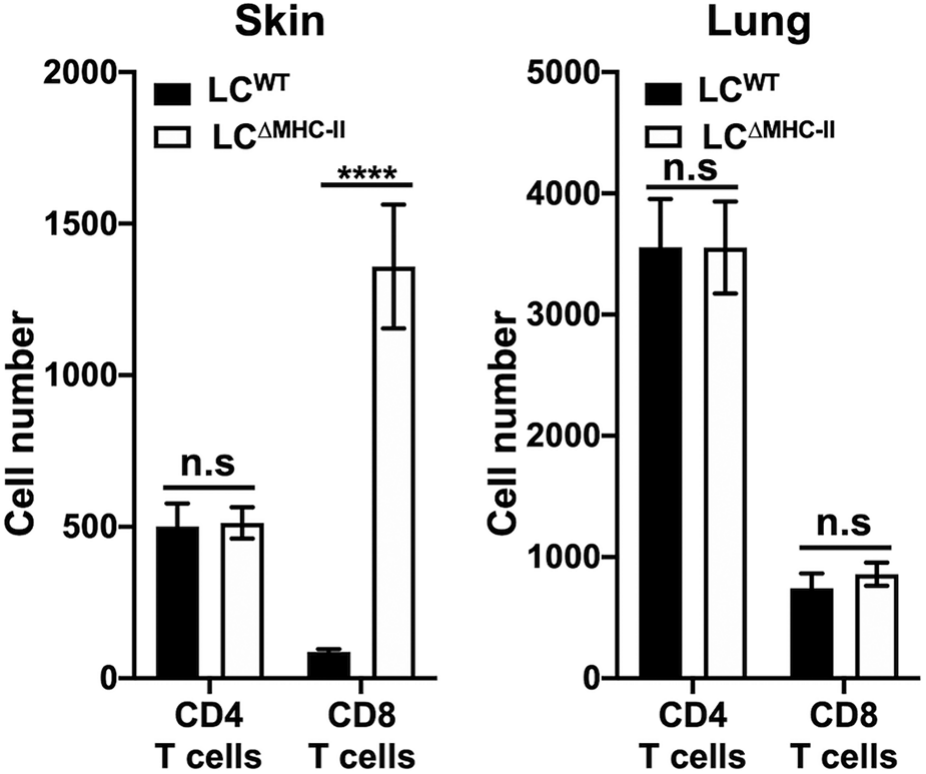
CD8 T cell numbers are increased in skin but not lungs of LC^ΔMHC-II^ mice. LC^ΔMHC-II^ mice or LC^WT^ littermate controls received 1.25 µg of rat anti-mouse CD45 conjugated to FITC intravenously 3 min prior to sacrifice to exclude blood-resident immune cells. Single cell-suspensions from left lung and ear skin peels were stained with rat anti-mouse mAbs to identify CD4 T cells and CD8 T cells by flow cytometry. Live interstitial CD8 T cells and CD4 T cells were identified as Zombie Aqua^lo^, CD45:FITC^−^, CD45:PE^+^, CD3^+^, TCRβ^+^, CD8α^+^, CD4^−^ and Zombie Aqua^lo^, CD45:FITC^−^, CD45:PE^+^, CD3^+^, TCRβ^+^, CD8α^−^ and CD4^+^, respectively. Numbers of CD4 T cells and CD8 T cells, normalized to 100,000 live non-immune cells, are presented for lung and skin tissues. Data are from 3 experiments with at least 8 mice per tissue type and are plotted with means ± SEM. Means were compared using an unpaired two-tailed Student's *t*-test. ****p < 0.0001, n.s., not significant.

Since CD8 T cells proximal to LCs are increased in the oral mucosa of LC^ΔMHC-II^ mice, we analyzed CD8 T cell numbers in other peripheral tissues where LCs reside. We hypothesize that LCs unable to display antigen on MHC-II molecules may be associated with the increase in CD8 T cells. To test this hypothesis, we compared lung, a mucosal tissue that does not ordinarily harbor LCs, with glabrous skin that possesses a LC-rich stratified epithelium (1). Congruent with our oral mucosa analysis (21), the number of CD4 T cells found in either the lung or the skin were similar when LC^ΔMHC-II^ mice were compared with LC^WT^ littermate controls, although CD4 T cells were about 7-fold more numerous in the lung than in skin (Figure 2). In contrast, CD8 T cells were significantly more numerous in the skin of LC^ΔMHC-II^ than LC^WT^ mice. In the same LC^ΔMHC-II^ mice, CD8 T cell numbers were not elevated in the lungs when compared to the LC^WT^ mice (Figure 2). We also examined the stratified epithelial cervical-vaginal tissue, which contains LCs (29), but the vascularity of the tissue and epithelial changes with different stages of estrus compromised the normalization of CD8 T cell numbers across samples based on the live non-immune cell population (data not shown).

### Oral mucosal CD8 T cells in LC^ΔMHC-II^ mice display an activated, tissue-resident memory phenotype and exhibit a broad TCR Vβ repertoire

**Figure 3:**
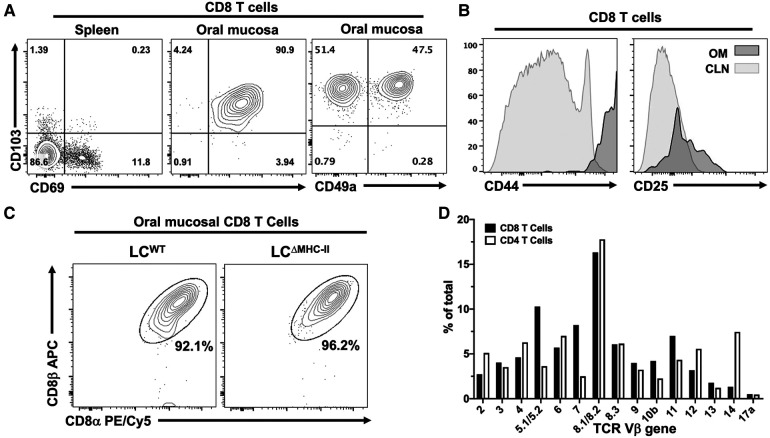
CD8 T cells in the oral mucosa of LC^ΔMHC-II^ mice have an activated tissue-resident memory phenotype, express the CD8 α/β heterodimer and have broad TCR Vβ usage. (**A**) Representative flow cytometry plots showing CD103, CD69 and CD49a expression on CD8 T cells isolated from tissues of a LC^ΔMHC-II^ mouse. Numbers indicate % of cells within each quadrant gate. (**B**) Representative flow cytometry histogram plots showing CD44 or CD25 expression on CD8 T cells isolated from cervical lymph nodes (CLN) or oral mucosa (OM) of a LC^ΔMHC-II^ mouse. (**C**) Representative flow cytometry plots showing CD8α and CD8β on CD8 T cells isolated from OM. Interstitial CD8 T cells were identified as Zombie Aqua^lo^, CD45:FITC^−^, CD45:PE^+^, CD3^+^, TCRβ^+^, CD4^−^. Cells were obtained from a single LC^ΔMHC-II^ animal or pooled from 4 LC^WT^ littermate mice. (**D)** Pooled cells from the epithelial sheets of 12 LC^ΔMHC-II^ mice were stained to identify CD4 T cells and CD8 T cells and subsequently stained with one of 15 different TCR Vβ mAbs. One aliquot remained unstained to set negative TCR-β gates for both CD4 T cells and CD8 T cells. CD4 T cells were identified as Zombie Aqua^lo^, CD45^+^, CD90.2^+^, CD3^+^, CD4^+^, CD8α^−^; CD8 T cells were identified as Zombie Aqua^lo^, CD45^+^, CD90.2^+^, CD3^+^, CD4^−^, CD8α^+^. The frequency of TCR Vβ usage amongst CD4 T cells and CD8 T cells is shown.

T cells in the periphery usually express cell surface markers characterizing them as either effector memory or tissue memory. CD69 is often used as a marker of memory and in conjunction with the alpha E integrin, CD103, has been used to distinguish tissue-resident memory T cells from effector memory T cells (30) including those T cells residing within the oral mucosa (31, 32). Tissue-resident Tc1 CD8 T cells are also marked by the alpha 1 integrin, CD49a (33, 34). To determine whether the oral CD8 T cells have an effector or tissue-resident memory phenotype, we analyzed expression of CD69 and CD103 on CD8 T cells isolated from the spleen and the oral mucosa of LC^ΔMHC-II^ mice. Additionally, we analyzed CD49a on oral CD8 T cells. The spleen provides a surrogate CD8 T cell comparison since LC^WT^ mice have too few CD8 T cells in the oral mucosa to analyze. We expected that the spleen would contain sufficient circulating effector memory cells (CD69^+ve^) to analyze but tissue-resident CD8 T cells (CD69^+ve^ CD103^+ve^) would be absent (Figure 3A—left panel). In the oral mucosa of LC^ΔMHC-II^ mice, greater than 90% of CD8 T cells are CD69^+ve^ CD103^+ve^ double positive, consistent with a tissue-resident memory phenotype (Figure 3A—center panel). CD103 expression is consistent with the epithelial location of the CD8 T cells (30). We also identified a CD103^+ve^ CD49a^+ve^ CD8 T cell population, but interestingly also an atypical CD103^+ve^ CD49a^−ve^ population at a similar frequency (Figure 3A—right panel).

Oral mucosa CD8 T cells from LC^ΔMHC-II^ mice were also examined for expression of two activation markers, CD44 and CD25 (Figure 3B). These CD8 T cells appeared highly activated, expressing uniformly elevated levels of CD44 compared to CD8 T cells from cervical lymph nodes. Compared to cervical lymph node CD8 T cells, CD25 expression was only slightly elevated in the oral mucosal population.

The MHC-I co-receptor, CD8, can exist as αα homodimers or αβ heterodimers. In mice, CD8αα T cells are predominately located within the mucosal epithelial lining of the small intestine (35). Given the mucosal residency of these CD8αα T cells, we asked whether CD8 T cells found in the oral mucosa of LC^ΔMHC-II^ also express the CD8αα homodimer (Figure 3C). In the oral mucosa of both LC^ΔMHC-II^ and LC^WT^ mice, however, CD8 T cells almost exclusively express the αβ heterodimer.

Mucosal-associated invariant T (MAIT) cells are another unconventional T cell subset that can express CD8 (36). Utilizing major histocompatibility complex I-related protein 1 (MR1)-specific tetramers, MAIT cells did not appear to reside in the CD8 T cell compartment of the oral mucosa of LC^ΔMHC-II^ mice (Supplementary Figure S2A). Nonetheless, CD4^−ve^ CD8^−ve^ MAIT cells were identified in small numbers within the oral mucosa of both LC^ΔMHC-II^ and LC^WT^ mice (Supplementary Figure S2B).

To explore the clonality of the CD8αβ T cells in the oral mucosa of LC^ΔMHC-II^ mice, we next examined their TCR Vβ usage. A limited repertoire of Vβ gene usage would indicate that the CD8 T cells recognized and have responded to a narrow range of antigens. The CD8 αβ T cell population appeared in contrast to be polyclonal, displaying a broad repertoire of TCR molecules with Vβ usage ranging from 16.33 to 1.33% for the clones tested (Figure 3D). As anticipated, TCR Vβ usage was also broad in the CD4 T cell compartment. Vβ gene 17a is not expressed in the C57BL/6J mouse and at <0.5% can be considered background detection.

### CD8 T cells in the oral mucosa of LC^ΔMHC-II^ mice express IL-17A

**Figure 4:**
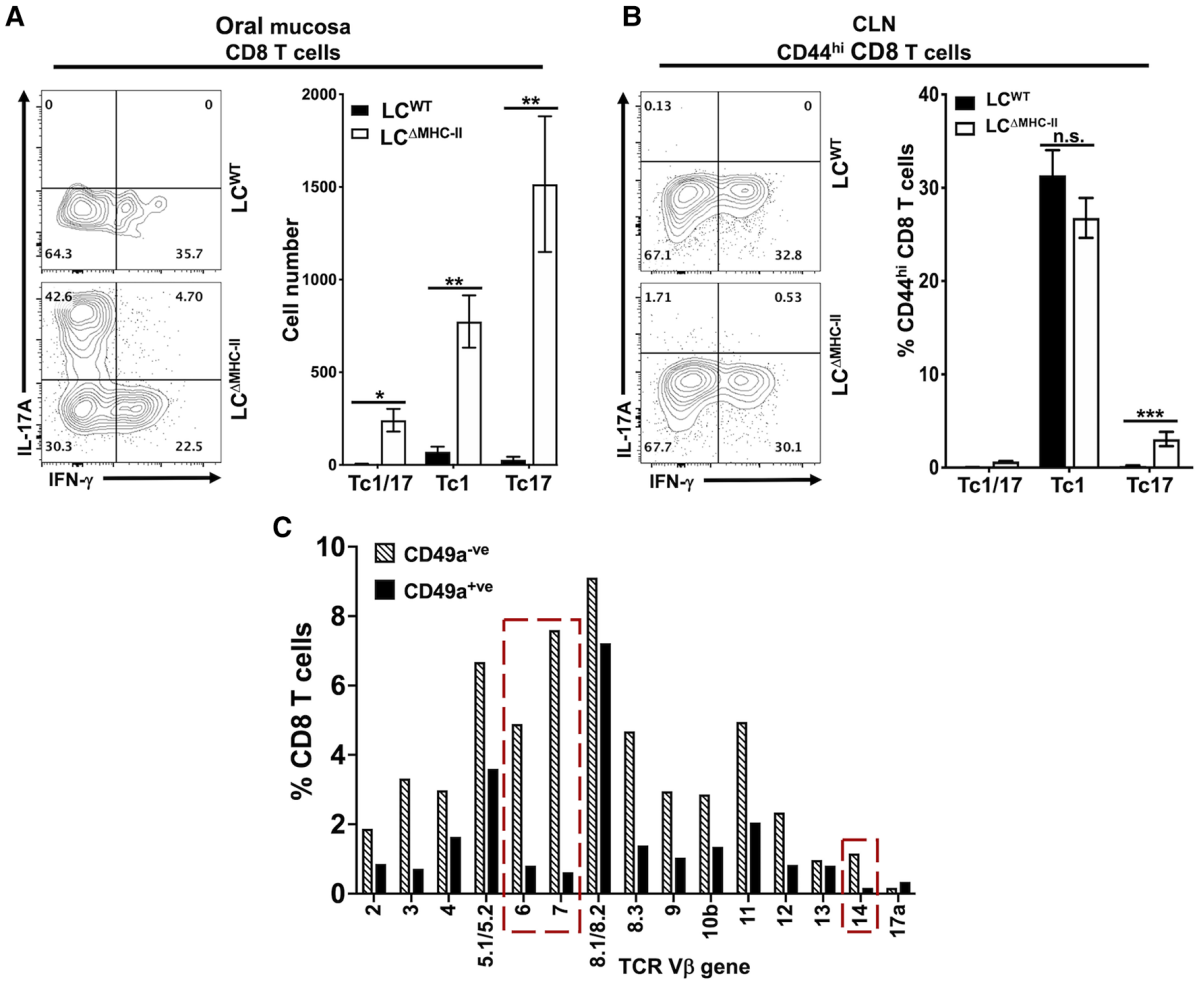
CD8 T cells in the oral mucosa of LC^ΔMHC-II^ mice predominately express IL-17A. Single cell suspensions were activated with PMA/ionomycin in the presence of brefeldin A, surface stained to identify CD8 T cells and then stained intracellularly with rat anti-mouse IL-17A and IFN-γ mAbs (A and B). Representative flow cytometry plots showing expression of IL-17A and IFN-γ are displayed. Numbers indicate % of cells within each quadrant gate. (**A**) Summary data from 3 independent experiments totaling at least 12 mice per group showing means ± SEM of Tc1 (IFN-γ^+^), Tc17 (IL-17A+) or Tc1/17 (IFN-γ^+^ and IL-17A^+^) CD8 T cell numbers normalized to 100,000 live non-immune cells. An unpaired two-tailed Student's *t*-test was used to test significance. *p < 0.05, **p < 0.01. (**B**) Summary data from 2 independent experiments of 8 mice per group showing means ± SEM of the relative frequency of Tc1, Tc17 and Tc1/17 CD8^+^ CD44^hi^ T cells in cervical lymph nodes. Unpaired Student's *t*-test was used to test significance. ***p < 0.001. n.s., not significant. (**C)** TCR Vβ usage was determined as described in Figure 3D. The frequency of TCR Vβ usage amongst CD49a^+^ and CD49a^−^ CD8 T cells is shown. Red dashed boxes highlight differences between CD49a^+^ and CD49a^−^ CD8 T cells of 5X or greater.

Classic Tc1 CD8 T cells typically express IFN-γ when IL-12 drives differentiation (37). However, the population of CD103^+ve^ CD49a^−ve^ CD8 T cells we identified (Figure 3A) is a phenotype associated with IL-17A expression (34). Consistent with our earlier published work (21), few CD8 T cells localize in the oral mucosa of LC^WT^ mice and these cells predominately expressed IFN-γ and not IL-17A (Figure 4A). In contrast, a plurality of CD8 T cells from LC^ΔMHC-II^ mice expressed IL-17A (Tc17), with smaller numbers expressing IFN-γ (Tc1) or both proinflammatory cytokines (Tc1/17) (Figure 4A). All three CD8 T cell phenotypes were significantly elevated in LC^ΔMHC-II^ compared to LC^WT^ mice. LC^ΔMHC-II^ mice show >30-fold more Tc17 cells than LC^WT^ mice. Interestingly, Tc1/17 cells numbers were also increased.

LC^ΔMHC-II^ mice could fail to differentiate robust numbers of Tc1 cells. To consider this possibility, we examined the phenotype of CD8 T cells found in the cervical lymph nodes, which drain the oral mucosa and head region. To exclude the majority naïve CD8 T cells, only CD8 T cells that displayed an activated memory (CD44^hi^) phenotype were analyzed (Figure 4B). In cervical lymph nodes, there was no significant difference in the frequency of Tc1 cells found in LC^ΔMHC-II^ mice and LC^WT^ mice. Both strains were found to have around 30% Tc1 cells, indicating that there is no defect in Tc1 cell generation in LC^ΔMHC-II^ mice. We did observe a small, but significant, increase in the frequency of Tc17 cells. The relative frequencies of Tc1 and Tc17 cells in cervical lymph nodes and in oral mucosa of LC^ΔMHC-II^ mice, however, are very different.

CD8 T cells in LC^ΔMHC-II^ mice generally displayed broad TCR Vβ usage (Figure 3D). CD8 T cells could also be separated into two distinct populations based on CD49a expression (Figure 3A). To determine differences in TCR Vβ usage between these two CD8 T cell populations, we used the anti-TCR Vβ mAb panel (Figure 4C). The Vβ genes 6, 7 and 14 showed frequencies that were at least 5-times greater amongst CD49a^−^ CD8 T cells than CD49a^+^ CD8 T cells, suggesting a differential response to antigens. Consistent with this observation and given the tight association of CD49a expression with Tc1 cells (33, 34), CD49a^+^ CD8 T cells were almost exclusively negative for IL-17A expression (data not shown).

### Intraepithelial CD4 T cells suppress CD8 T cell numbers in the oral mucosa

**Figure 5:**
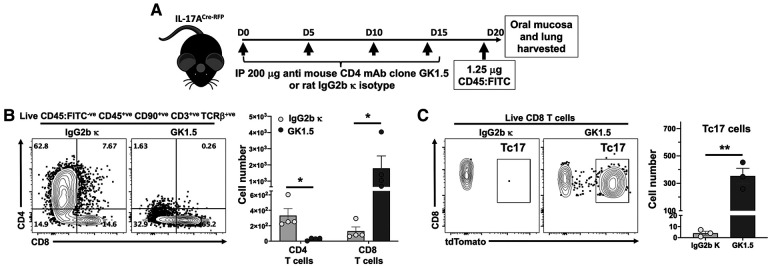
Depletion of CD4 T cells results in expansion of both CD8 T cells and Tc17 in the oral mucosa. (**A**) Experimental design to deplete CD4 T cells. 8–10 weeks old IL-17A^Cre-RFP^ mice were injected i.p. with 200 µg anti-CD4 mAb (Clone GK1.5; BioXCell) or Rat IgG2b *κ* control (#BE0090; BioXCell) 4 times, 5 days apart. On day 20 (D20) mice were intravenously injected with FITC-labeled anti-CD45 mAb and sacrificed 3 min later. Oral mucosa epithelial peels were treated with Dispase II, collagenase D and DNAse I and single cell suspensions stained to identify CD4 T cells and CD8 T cells by flow cytometry. (**B)** Typical flow cytometry plots showing live oral mucosa interstitial CD4 T cells and CD8 T cells from a GK1.5 or Rat IgG2b *κ* treated animal. Summary data shows mean ± SEM of CD4 T cell and CD8 T cell numbers. (**C)** Typical flow cytometry plots showing tdTomato expression (IL-17A proxy) in live oral mucosal interstitial CD8 T cells from a GK1.5 or Rat IgG2b *κ* treated animal. Mean ± SEM of Tc17 cells is plotted. Summary data compared by Student's two-tailed *t*-test. *p < 0.05; **p < 0.01.

Given the effect of MHC-II presentation by LCs on intraepithelial CD8 T cell numbers, we tested the contribution of CD4 T cells on the control of CD8 T cell numbers in LC MHC-II competent mice. Global ablation of CD4 T cells in IL-17A^Cre-RFP^ reporter mice (Figure 5A) with an anti-CD4 mAb (38–40) resulted in increased intraepithelial CD8 T cell numbers by >14-fold (Figure 5B). Interestingly, 25%–66% of these CD8 T cells in the oral mucosa expressed IL-17A (Tc17) as measured by proxy tdTomato expression (Figure 5C). In contrast, CD8 T cells in the lungs of these mice did not increase or express IL-17A despite a >99% reduction in CD4 T cells (data not shown).

### γδ T cells correlate with CD8 T cell numbers and are suppressed in the oral mucosa of LC^ΔMHC-II^ mice

**Figure 6:**
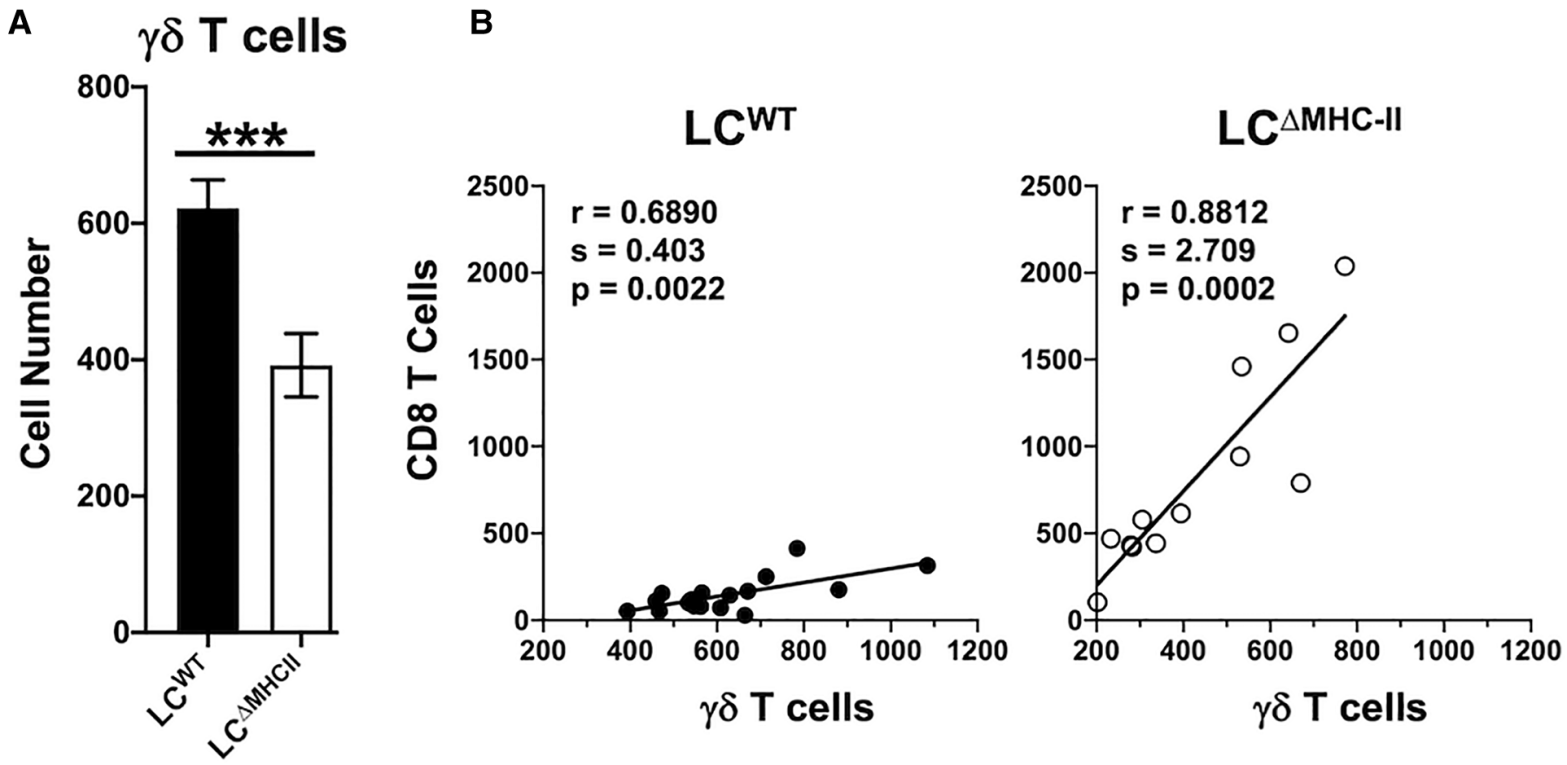
Decreased γδ T cells in the oral mucosa of LC^ΔMHC-II^ mice correlate with CD8 T cell numbers. (**A**) Oral mucosa from LC^ΔMHC-II^ mice or LC^WT^ littermate controls was processed, and single cell suspensions stained with rat anti-mouse mAbs to identify γδ T cells by flow cytometry. Live γδ T cells were identified as Zombie Aqua^lo^, CD45^+^, CD3^hi^, TCRγ/δ^+^, TCRβ^−^, CD8α^−^, CD4^−^ cells. Summary data of γδ T cell numbers, normalized to 100,000 live non-immune cells, is presented. Data are from 3 independent experiments with at least 12 mice per group are plotted as means ± SEM. Means were analyzed using an unpaired two-tailed Student's *t*-test. ***p < 0.001. (**B**) Correlation between CD8 T cells and γδ T cells numbers identified in oral mucosal tissue of LC^ΔMHC-II^ mice or LC^WT^ littermate controls by flow cytometry as described in A. Data are from 3 independent experiments with at least 12 mice per group. Simple Linear Regression was utilized to fit the slope(s) to the data. The significance (*p* value), of the calculated Pearson correlation coefficient (r) is reported.

CD4 T cell abundance and Th17 frequency are not altered in the oral mucosa of LC^ΔMHC-II^ mice compared to LC^WT^ mice (21). Given the increase in LC^ΔMHC-II^ Tc17 cells, we asked whether γδ T cells, which primarily express IL-17A, are also increased. Although the percentage of γδ T cells expressing IL-17A was ∼80% in both LC^ΔMHC-II^ and LC^WT^ mice (data not shown), the number of oral mucosal γδ T cells expressing IL-17A was lower in LC^ΔMHC-II^ mice (Figure 6A).

CD8 T cell and γδ T cell numbers seemed differentially impacted in the oral mucosa of LC^ΔMHC-II^ mice. We next sought a relationship between CD8 T cell and γδ T cell numbers. Cell numbers were normalized across samples to live non-immune cells (primarily keratinocytes) to control for differences in sample processing (41). Interestingly, despite the low number of γδ T cells in the oral mucosa of LC^ΔMHC-II^ mice, CD8 T cell and γδ T cell numbers showed a positive and statistically significant correlation (Figure 6B). A positive correlation was also seen in the oral mucosa of LC^WT^ mice, albeit with a smaller slope value than in LC^ΔMHC-II^ mice.

### Expression of TGF-β1 is significantly elevated in the oral mucosa of LC^ΔMHC-II^ mice

**Figure 7:**
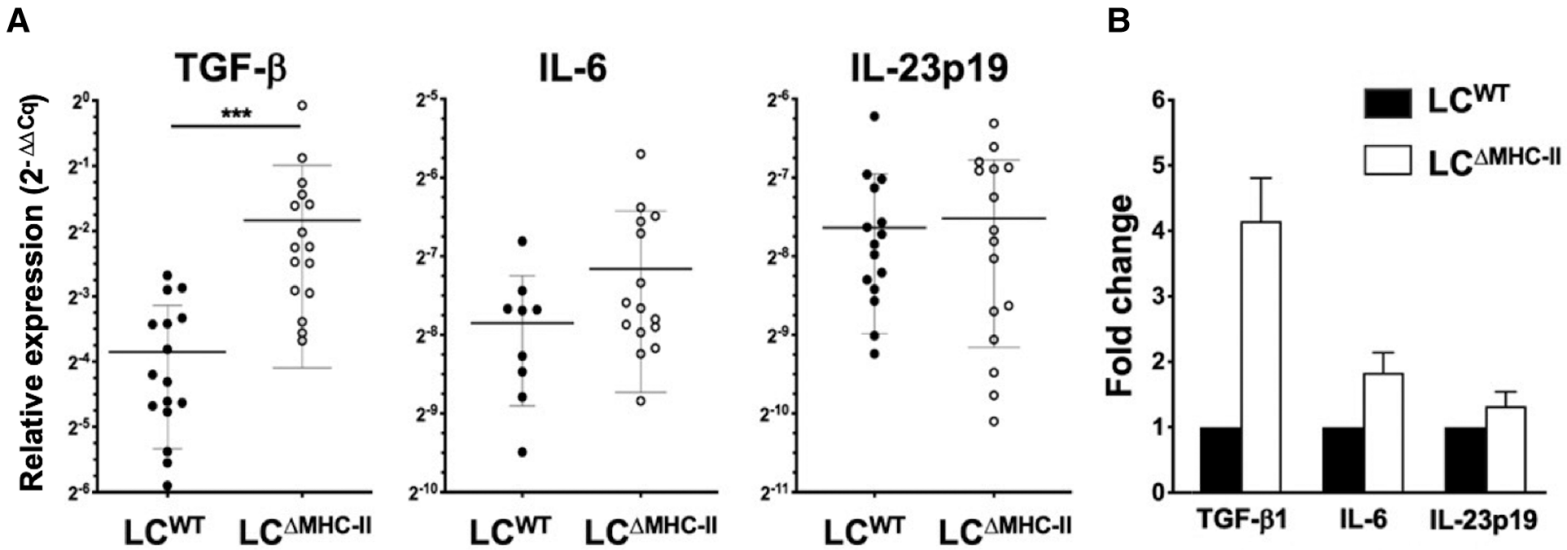
Expression TGF-β1 is significantly elevated in the oral mucosa of LC^ΔMHC-II^ mice. Total RNA extracted from the oral mucosa of age-matched (8–9 week-old) LC^ΔMHC-II^ mice or LC^WT^ littermate controls was converted into cDNA and qPCR analysis performed. The ΔΔCq method was used to analyze the raw qPCR data (Cq values) following normalization to expression of the housekeeping gene, hydroxymethylbilane synthase. (**A**) Summary data of relative TGF-β1, IL-6 and IL-23p19 mRNA expression levels. Individual data points are plotted along with mean ± SEM. Data is from three independent experiments involving at least 9 mice. A two-tailed Student's *t*-test was used to test significance. ***p < 0.001; ****p < 0.0001. (**B**) Relative fold changes of TGF-β1, IL-6, and IL-23p19 mRNA in oral mucosal samples from LC^ΔMHC-II^ and WT mice.

Tc17 and Th17 cells have similar IL-6 and TGF-β1 requirements to drive polarization from naïve T cells in lymph nodes or maintenance and clonal expansion in the periphery (42–44). Furthermore, Tc17 cells maintain all the transcriptional circuitry needed to convert to a Tc1 phenotype where sustained IL-6-driven expression of Signal Transducer and Activator of Transcription 3 (STAT3) and Retinoic acid-related Orphan Receptor gamma *t* (RORγt) overrides the Tc1 program (42). We used qPCR analysis to examine whether LC^ΔMHC-II^ mice have elevated IL-6 or TGF-β1 expression in oral mucosa (Figure 7A). Here, we found that TGF-β1 gene expression was 4-times higher than LC^WT^ mice, while IL-6 gene expression was similar in the two strains (Figure 7B).

Given increased levels of TGF-β1 expression in the oral mucosa, we extended the analysis to a broader set of genes that might be involved in sustaining Tc17 and Tc1 cells. Specifically, the global expression of IL-23p19 (Figure 7A), IL-12p35 and IFN-γ (data not shown) was similar in the two strains. Gene expression of IL-21 and IL-17A was not detected (Cq > 35) in the oral mucosa of either mouse strain. The global expression of the Treg-associated genes, IL-10 and EBI-3 were also similar in the oral mucosa of LC^ΔMHC-II^ and LC^WT^ mice (data not shown).

### TGF-β1 signaling is not required to maintain oral Tc17 cells in LC^ΔMHC-II^ mice

**Figure 8:**
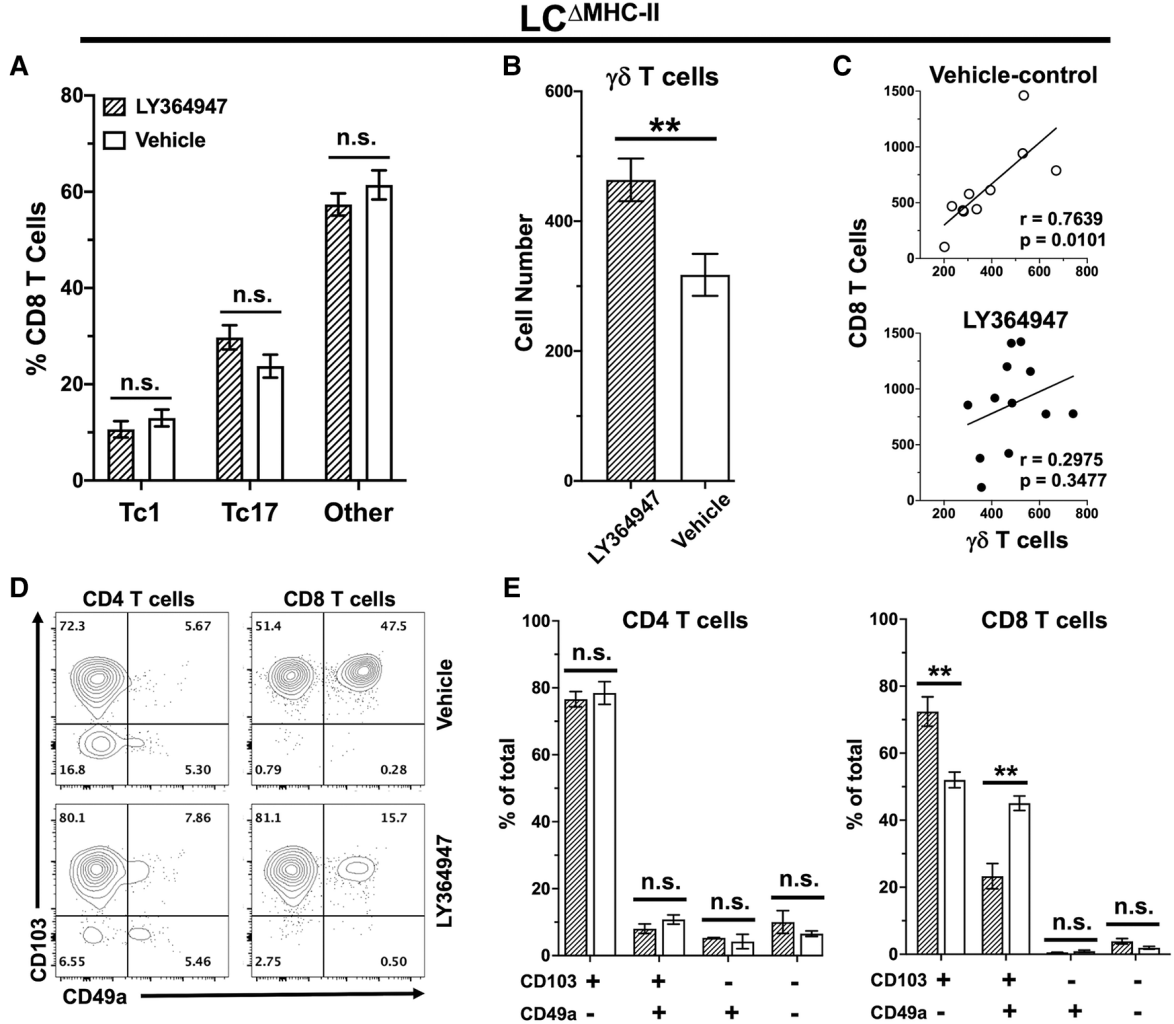
TGF-R1 kinase inhibition impacts γδ T cells and CD49a^+^ CD8 T cells but not Tc17 in LC^ΔMHC-II^ mice. LC^ΔMHC-II^ mice received vehicle control (2% DMSO in PBS) or 4 mg/kg LY364947 (2% DMSO in PBS) intraperitoneally daily for 7 days. Single cell suspensions from oral mucosa were analyzed by flow cytometry. (**A**) Summary data showing mean frequency ± SEM of Tc1, Tc17 and IL-17A/IFN-γ non-expressing CD8 T cells (other). Data are from 2 independent experiments with at least 8 mice per group. Means were analyzed using unpaired two-tailed Student's *t*-test. **p < 0.01, n.s., not significant. (**B**) Summary data from 3 independent experiments with at least 10 mice per treatment group showing mean number ± SEM of γδ T cells. Live γδ T cells were Zombie Aqua^lo^, CD45^+^, CD3^hi^, TCR γ/δ^+^, TCRβ^−^, CD8α^−^, CD4^−^. Cell number was normalized to 100,000 live non-immune cells. Means were compared by unpaired two-tailed Student's *t*-test. (**C**) Correlation between CD8 T cell and γδ T cell numbers. Simple linear regression was used to fit the best slope to the data. Significance (*p* value) of the calculated Pearson correlation coefficient (r) is shown. (**D**) Representative flow plots showing CD103 and CD49a on oral mucosal CD4 and CD8 T cells following LY364947 or vehicle treatment. (**E**) Summary data showing frequency of CD103 and CD49a on CD4 or CD8 T cells. Data are from 3 to 4 mice. Means were analyzed by unpaired two-tailed Student's *t*-test. **p < 0.01, n.s., not significant.

TGF-β1 is essential *in vitro* to differentiate naïve CD8 T cells towards a Tc17 phenotype (17, 45). To investigate the *in vivo* requirement for TGF-β1 in Tc17 differentiation and/or maintenance, we disrupted TGF-β-dependent signaling in LC^ΔMHC-II^ mice with the kinase inhibitor LY364947 (26). LY364947 treatment did not alter the frequency of Tc17 cells in the oral mucosa when compared to mice treated with vehicle control (Figure 8A). Additionally, LY364947 treatment did not impact the frequencies of Tc1 cells or CD8 T cells not expressing IL-17A or IFN-γ.

We also questioned whether TGF-β1 signaling might be involved in the decreased numbers of γδ T cells in the oral mucosa of LC^ΔMHC-II^ mice (Figure 6A). Inhibition of TGF-β1 signaling with LY364947 significantly increased the number of γδ T cells in LC^ΔMHC-II^ mice (Figure 8B). TGF-β1 signaling appears to selectively impact γδ T cells and not CD8 T cells since the correlation between γδ T cell and CD8 T cell numbers in the oral mucosa is maintained in the vehicle control group but is lost in mice treated with LY364947 (Figure 8C).

The expression of CD49a and CD103 in CD8 T cells is reported to depend on TGF-β1 signaling (46, 47). Here, we determined the frequency of CD49a and CD103 expression on both CD4 T cells and CD8 T cells isolated from the oral mucosa of LC^ΔMHC-II^ mice treated with LY364947 or the vehicle control (Figure 8D). Inhibition of TGF-β1 signaling with LY364947 did not impact CD4 T cells (Figure 8E). However, in the CD103^+ve^ subpopulation of CD8 T cells there was a significant decrease in the frequency of CD49a^+ve^ cells and a concomitant increase in the frequency of the CD49a^−ve^ cells in LY364947 treated LC^ΔMHC-II^ mice indicating that CD49a expression, but not CD103, was sensitive to TGF-β1 signaling disruption (Figure 8E). Moreover, the MFI of the CD103 signal remained unchanged (data not shown).

### LC^ΔMHC-II^ mice have enhanced resistance to oral *Candida albicans* infection

**Figure 9:**
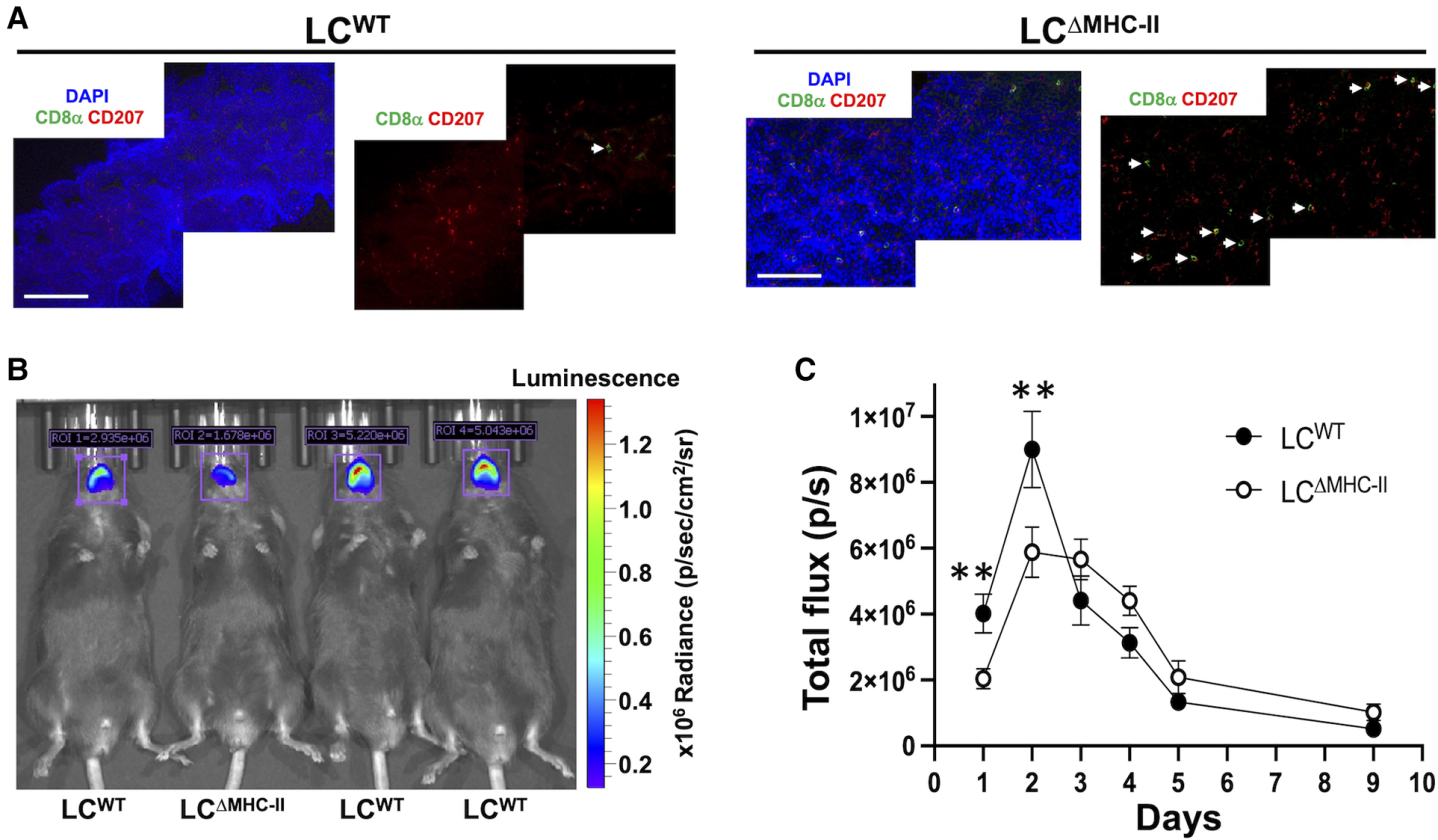
LC^ΔMHC-II^ mice have enhanced CD8 T cell numbers in tongue epithelium and increased resistant to oral candidiasis. (**A**) Epithelial sheets from tongues of LC^WT^ and LC^ΔMHC-II^ mice were stained with DAPI (blue), rat anti-mouse CD8α mAb (green) and rat anti-mouse CD207 mAb (red). Representative confocal microscope images (40×) obtained from a LC^ΔMHC-II^ mouse and a LC^WT^ are shown. White arrows indicate CD8α^+^ cells. Scale bar is 100 µm. (**B,C**) Age matched male sibling mice were co-infected with *S. oralis* 34 and *C. albicans* strain DSY4976 that expresses a red-shifted firefly luciferase gene. Mice were not immunosuppressed. Bioluminescence was measured on days 1, 2, 3, 4, 5, and 9 following oral administration of D-Luciferin. (**B**) Superimposed photograph with detected bioluminescence captured on day 1 using the IVIS Spectrum *in vivo* imaging system. Regions of interest (ROI) used to quantify bioluminescence are indicated along with the total flux in photons per s detected within the ROI. (**C**) Summary data from 4 independent experiments showing bioluminescence (total flux p/s) detected in the oral cavity of mice over 9 days. *N* = 16 (LC^WT^) and *N* = 13 (LC^ΔMHC-II^). Mann–Whitney *U*-test was used to compare ranks at each day. **p < 0.01.

Innate and adaptive immunity to *C. albicans* is significantly compromised at mucosal surfaces when IL-17A or its signaling pathway are abrogated (8, 14). IL-17A contributes to *C. albicans* resistance by inducing epithelial cells to express antimicrobial peptides and neutrophil chemotactic chemokine CXCL8 and GM-CSF (13). *C. albicans* in the murine oral cavity typically shows tropism for the dorsal surfaces of the tongue, therefore, we compared the number of LCs and CD8 T cells in the dorsal epithelial sheets of LC^ΔMHC-II^ and LC^WT^ (Figure 9A). Despite similar number of LCs, the number of CD8α^+ve^ cells were significantly higher in LC^ΔMHC-II^ mice.

Given the increased numbers of Tc17 cells, we tested LC^ΔMHC-II^ mice for resistance to oral infection with *C. albicans*. We utilized an established *C. albicans* and *Streptococcus oralis* co-infection model that avoids the confounding effects of artificially induced immunosuppression to establish oral infection (27). For this work, we employed a *C. albicans* strain engineered to express a red-shifted firefly luciferase gene and measured *in vivo* the emitted bioluminescence as a proxy for *C. albicans* biomass over time (28) (Figure 9B). Although the numbers of Tc17 in the oral mucosa of male and female LC^ΔMHC-II^ mice are similar, we found that male mice of either strain were significantly more susceptible to *C. albicans* infection than their female counterparts (data not shown). Therefore, we elected to work with male mice in further experiments. While the burden for both mouse strains peaked at day 2, LC^ΔMHC-II^ mice had significantly less *C. albicans* burden than LC^WT^ mice at day 1 and day 2 time points (Figure 9C). Interestingly, LC^WT^ mice apparently cleared *C. albicans* faster than LC^ΔMHC-II^ mice from day 2 to day 3. However, from day 3 until the end of the experiment at day 9 *C. albicans* decreased at a similar rate in both mouse strains.

## Discussion

In the oral mucosa of LC^ΔMHC-II^ mice engineered to develop LCs unable to express MHC-II we discovered strikingly high numbers of CD8 T cells (21). This work characterizes those CD8 T cells as intraepithelial tissue-resident CD8αβ T cells, the majority of which express IL-17A and not IFN-γ. These characteristics marks them as atypical Tc17 cells. In addition, the intraepithelial CD8αβ T cells are not mucosal-associated invariant T cells (MAIT cells) because they not do not recognize MR1 tetramers. Given the importance of IL-17A to mucosal immunity, particularly against *C. albicans* (8, 10, 13, 14), we also determined that LC^ΔMHC-II^ mice displayed enhanced resistance to oropharyngeal candidiasis.

A global MHC-II knockout mouse has been available for over 20 years (48), and the associated defect in the CD4 T cell compartment is well characterized. Perhaps not surprising, relatively few studies have characterized these mice for defects in the CD8 T cell compartment (49–51). In a model of human papillomavirus induced carcinoma, increased CD8 T cell numbers found in carcinomas of MHC-II knockout mice contributed to better control of tumor growth (51). Whether these cells were Tc1 or Tc17 was not determined, but the authors speculated that increased CD8 T cells were a compensatory effect for reduced numbers of tumor-infiltrating Th1 cells. In contrast, although oral Th1 and Helios^+^ Treg CD4 T cell compartments are reshaped, Th17 cell number is not impacted in LC^ΔMHC-II^ mice (21). This finding indicates that increased Tc17 cells are not a compensation for a loss in Th17 cell numbers. Interestingly, Th17-mediated protection against *Citrobacter rodentium* intestinal infections is abrogated in a bone marrow chimeric mouse model where MHC-II presentation is absent only on hematopoietic cells (50). Coincidentally, the number of Tc1 and Tc17 cells found in the gut of these mice was substantially increased. Unexpected expansion or control of Tc17 cells has also been described in situations of both CD4 T cell deficiency and the complete absence of CD4 T cell help (19, 52–56). CD4 Treg cells play an active role suppressing CD8 T cells either through mechanisms involving local MHC-II antigen presentation or through T cell receptor-independent processes (57). Certainly, our CD4 T cell ablation data supports the notion that a CD4 T cell population, possibility CD4 Treg cells, are regulating CD8 T cells via LCs in tissues where LCs, CD4 T cells and CD8 T cells co-exist. This contention is bolstered by data that showed CD8 T cell numbers in LC^ΔMHC-II^ mice were also dramatically increased in LC-rich tissue such as skin but not in the lung where LCs are not usually found. In addition, we found no increase in CD8 T cell numbers in lung despite near complete ablation of CD4 T cells.

CD8 T cells are a minority CD3^+^ T cell in the oral mucosa of humans and mice at steady state (21, 31, 58). Unlike gut intraepithelial CD8 T cells, most oral CD8 T cells that express CD69 are negative for CD103 indicating that they are likely recirculating effector memory T cells and not bona fide residents of the oral mucosa (31, 58). In stark contrast to these findings, greater than 90% of CD8 T cells in the oral mucosa of LC^ΔMHC-II^ mice co-expressed CD69 and CD103. Together with uniformly high CD44 expression and their intraepithelial location, these cells have the signature of conventional tissue-resident memory CD8 T cells (30). Furthermore, the relative paucity of Tc17 cells over Tc1 cells in the cervical lymph nodes that drain the oral mucosa suggests that most Tc17 cells generated in the LC^ΔMHC-II^ mouse do not enter the recirculating effector memory population.

TGF-β1 was significantly elevated in the oral mucosa of LC^ΔMHC-II^ mice and could potentially contribute to supporting the expansion of CD8 T cells. TGF-β signaling has been shown to be important for *in vitro* expression of CD103 on CD8 T cells and the *in vivo* maintenance of CD103^+^ intraepithelial gut resident CD8 T cells (59) and tissue-resident CD8 T cells in the skin (60). When inhibiting TGF-β signaling with LY364947 *in vivo*, the surface expression of CD103 on T cells and the frequency of CD103^+^ CD8 T cells did not change in the oral mucosa of LC^ΔMHC-II^ mice. Several factors may explain the apparent discrepancy between our data and that of Mackay et al. (59). First, there may be a different TGF-β threshold requirement for CD103 expression by CD8 T cells that ultimately migrate to and then take up residency in the gut or oral mucosa. Second, we used pharmacological intervention and not a genetic model to disrupt TGF-β signaling. In considering these discrepancies, it is also important to reiterate that TGF-β1 expression was elevated in the oral mucosa of LC^ΔMHC-II^ mice.

Consistent with the finding that CD49a expression on Tc1 cells *in vitro* appears to be dependent on TGF-β (46), our *in vivo* data reveals a significant reduction in the frequency of intraepithelial CD49a^+^ Tc1 cells in the oral mucosa of LC^ΔMHC-II^ mice. When TGF-β1 signaling was genetically abrogated or neutralized in a model of graft vs. host disease, the frequency of Tc17 cells did not change in agreement with our LY364947 treatment findings (61, 62). Taken together, we contend that oral intraepithelial CD8 T cells, and specifically Tc17 cells in LC^ΔMHC-II^ mice do not require TGF-β for maintenance of tissue residency.

Tc17 cells can contribute to inflammatory diseases, are associated with poor prognosis in some cancers, but also contribute to repair at barrier tissues like the skin, and via specific vaccines offer resistance to viral, bacterial and fungal pathogens (63). Unlike in psoriasis where Tc17 cell numbers are increased (33, 64), LC^ΔMHC-II^ mice do not exhibit pathological changes at oral mucosal surfaces or skin despite the high numbers of Tc17. Tc17 cells in LC^ΔMHC-II^ mice, therefore, do not seem to be overtly pathogenic. In this respect, LC^ΔMHC-II^ mice are similar to wild-type mice exposed to the commensal *Staphylococcus epidermidis* where antigen-specific skin resident Tc1 and Tc17 cell numbers are boosted without an associated inflammatory response (18, 65). Interestingly, exposure of skin to *S. epidermidis* offered prophylactic protection against *C. albicans* in a murine dermal infection model that was directly attributable to IL-17A expressed by Tc17 cells (18). Barrier resistance to *C. albicans* at both mucosal and skin surfaces critically depends on IL-17A promoting expression of antimicrobial peptides (12, 15) and eliciting recruitment of *C. albicans*-killing neutrophils (3, 10, 13, 14, 66, 67). Although TCR-independent bystander activation (68) cannot be excluded, IL-17A expression and protection against *C. albicans* in LC^ΔMHC-II^ mice could be driven by the presentation of antigens derived from members of the oral microbial community through TCR cognate interaction. Skin resistance to *C. albicans* infection induced by *S. epidermidis* seems to support a mechanism independent of *C. albicans* antigens (18). Broad TCR Vβ gene usage by the CD8 T cells is consistent with this viewpoint. Regardless of the mechanism, resistance to *C. albicans* in LC^ΔMHC-II^ mice warrants further investigation.

The oral mucosa is typically quiescent despite constant exposure to antigens derived from ingested food and a shedding oral microbiome. CD4^+^ FoxP3^+^ Treg cells comprise 30%–50% of the CD4 T cells found in the mouse oral mucosa at steady state, potentially implicating Treg cells in keeping this tissue quiescent (21, 69). In cancer, antigens presented by intratumoral dendritic cells drive Treg cells to actively suppress CD8 T cells (57, 70). Reduced MHC-II antigen presentation capacity by LCs in LC^ΔMHC-II^ mice might blunt the suppressive function of oral Treg cells via immunosuppressive cytokines IL-10 and/or IL-35 (71–73). Any reduction in local immunosuppression may, therefore, allow the expansion of tissue-resident CD8 T cells.

In summary, we report here that reduced frequency of oral LCs expressing MHC-II results in increased intraepithelial tissue-resident Tc17 cells in oral mucosa. Although to a lesser extent, classic IFN-γ expressing Tc1 cells also increased. Manipulating MHC-II dependent interactions with LCs in barrier tissues could be exploited to boost defense against diseases that benefit from increased Tc1 or Tc17 responses. Further work is required to delineate the exact mechanism by which LCs dampen CD8 T cells via CD4 T cells at barrier tissues. Here it would be of interest to determine if CD8 T cells in the oral mucosa respond to stable members of the oral microbial community and whether these CD8 T cells recognize formylated peptides presented on non-classical MHC-I molecules (65).

## Data Availability

The original contributions presented in the study are included in the article/**Supplementary Material**, further inquiries can be directed to the corresponding author.

## Supplementary material

Supplementary Figure S1 Identification of immune cell subsets in mouse oral mucosa by flow cytometry. Flow cytometry gating strategy utilized to identify immune cell subsets in total oral mucosa, epithelial sheets from oral mucosa, or lamina propria from oral mucosa. Single cell suspensions from an oral mucosal epithelium sheet from a LC∆MHC-II mouse were stained with Zombie Aqua to distinguish live from dead cells, Fc receptors blocked with rat anti-mouse CD16/32 mAb followed by staining with a panel of rat anti-mouse mAbs against cell surface markers. Typical flow cytometry plots obtained are shown. Percentage of cells within specific gates are indicated. The population of live non-immune cells used for normalization purposes is indicated.

Supplementary Figure S2 IL-17A expressing CD8 T cells in LC∆MH-II mice are not MAIT cells. Oral mucosa was harvested from LC∆MH-II mice or pooled from LCWT littermate controls. Single cell suspensions were stained with MR1 tetramers MR1 5-OP-RU or MR1 6-FP and then surface stained to identify CD8 T cells, CD4 T cells and CD3+ double negative (DN) T cells. Live CD8 T cells were identified as Zombie Aqualo, CD45+, CD3+, TCRb+, CD8a+, CD4-; Live CD4 T cells as Zombie Aqualo, CD45+, CD3+, TCRb+, CD8a-, CD4+; Live CD3+ DN T cells as Zombie Aqualo, CD45+, CD3+, TCRb+, CD8a-, CD4-. (**A**) Representative flow cytometry dot plots for CD4 T cells (left panels) and CD8 T cells (right panels) for both the MAIT MR1 tetramer (MR1 5-OP-RU) and the MR1 negative control tetramer (MR1 6-FP). Gates, drawn to detect potential MAIT cells, are based off the negative control samples stained with MR1 6-FP. Numbers indicate percentage of cells within the drawn gates. (**B**) Representative flow cytometry dot plots for MR1 5-OP-RU and MR1 6-FP stained CD3+ DN T cells.
