# Supplementary material for: MHC-II presentation by oral Langerhans cells impacts intraepithelial Tc17 abundance and Candida albicans oral infection via CD4 T cells
Source: Front Oral Health. 2024 May 30;5:1408255. doi: 10.3389/froh.2024.1408255 (PMC11169704; doi:10.3389/froh.2024.1408255)
Supplement: Supplementary file 1 [file Table1.pdf]

Supplementary Table 1

Gene	Primers[a]	qPCR cDNA amplicon	Intron flanked
il17a	Fwd 5' CAG ACT ACC TCA ACC GTT CCA C 3' (257-278)<br>Rev 5' TCC AGC TTT CCC TCC GCA TTG A 3' (365-386)	129 bp	Intron 2
Tgfb1	Fwd 5' CCT GAG TGG CTG TCT TTT GAC G 3' (1444-1465)<br>Rev 5' AGT GAG CGC TGA ATC GAA AGC 3' (1514-1534)	90 bp	Intron 3
il6	Fwd 5' AAG AGA CTT CCA TCC AGT TGC CTT C 3' (96-120)<br>Rev 5' ATT ATA TCC AGT TTG GTA GCA TCC ATC 3' (367-393)	297 bp	Intron 1
il23a	Fwd 5' CCA GCG GGA CAT ATG AAT CT 3' (260-279)<br>Rev 5' AGG CTC CCC TTT GAA GAT GT 3' (435-454)	194 bp	Intron 1/2
il21	Fwd 5' TCA GCT CCA CAA GAT GTA AAG GG 3' 234-256)<br>Rev 5' GGG CCA CGA GGT CAA TGA T 3' (336-354)	120 bp	Intron 2
il10	Fwd 5' CAG AGC CAC ATG CTC CTA GA 3' 176-195)<br>Rev 5' GTC CAG CTG GTC CTT TGT TT 3' (234-253)	77 bp	Intron 1
Ebi3	Fwd 5' AGA GCC ACA GAG CAT GTC CAA 3' (227-247)<br>Rev 5' TGC ACT CTG GGC TGG CTT AG 3' (318-337)	110 bp	Intron 1
il12a	Fwd 5' CCG GTC CAG CAT GTG TCA A 3' (19-37)<br>Rev 5' CAG GTT TCG GGA CTG GCT AAG A 3' (127-148)	129 bp	Intron 1
ifng	Fwd 5' GCC ATC AGC AAC AAC ATA AGC G 3' (365-386)<br>Rev 5' GGG TTG TTG ACC TCA AAC TTG G 3' (459-480)	115 bp	Intron 3
Hmbs	Fwd 5' GAG TCT AGA TGG CTC AGA TAG CAT GC 3' (1035-1060)<br>Rev 5' CCT ACA GAC CAG TTA GCG CAC ATC 3' (1261-1284)	249 bp	Intron 13

[a] Bracketed numbers indicate predicted annealing position of primers on cDNA
