# Supplementary figures and images for: MHC-II presentation by oral Langerhans cells impacts intraepithelial Tc17 abundance and Candida albicans oral infection via CD4 T cells
Source: Front Oral Health. 2024 May 30;5:1408255. doi: 10.3389/froh.2024.1408255 (PMC11169704; doi:10.3389/froh.2024.1408255)

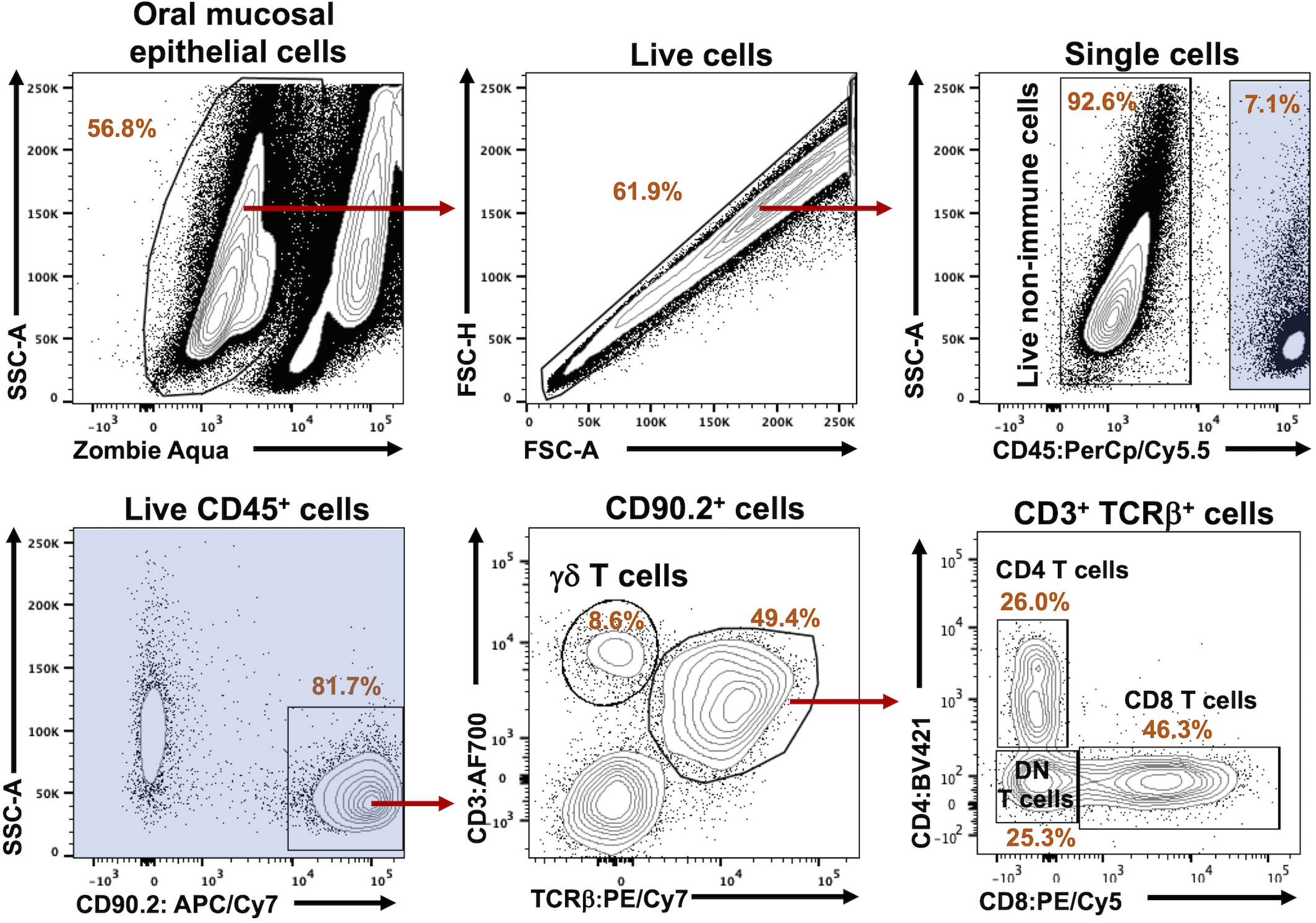

Supplement: Supplementary Figure S1 — Identification of immune cell subsets in mouse oral mucosa by flow cytometry. Flow cytometry gating strategy utilized to identify immune cell subsets in total oral mucosa, epithelial sheets from oral mucosa, or lamina propria from oral mucosa. Single cell suspensions from an oral mucosal epithelium sheet from a LC∆MHC-II mouse were stained with Zombie Aqua to distinguish live from dead cells, Fc receptors blocked with rat anti-mouse CD16/32 mAb followed by staining with a panel of rat anti-mouse mAbs against cell surface markers. Typical flow cytometry plots obtained are shown. Percentage of cells within specific gates are indicated. The population of live non-immune cells used for normalization purposes is indicated. [file Image1.tiff]

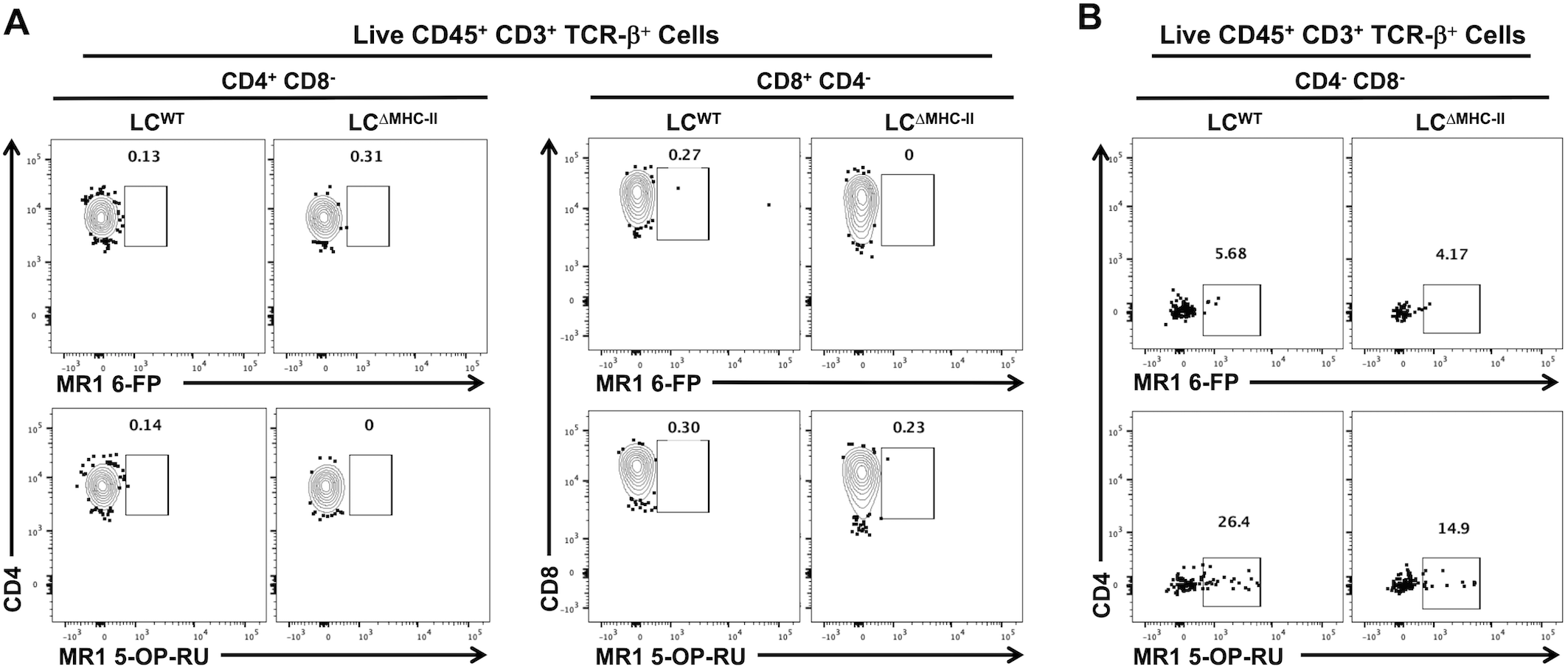

Supplement: Supplementary Figure S2 — IL-17A expressing CD8 T cells in LC∆MH-II mice are not MAIT cells. Oral mucosa was harvested from LC∆MH-II mice or pooled from LCWT littermate controls. Single cell suspensions were stained with MR1 tetramers MR1 5-OP-RU or MR1 6-FP and then surface stained to identify CD8 T cells, CD4 T cells and CD3+ double negative (DN) T cells. Live CD8 T cells were identified as Zombie Aqualo, CD45+, CD3+, TCRb+, CD8a+, CD4-; Live CD4 T cells as Zombie Aqualo, CD45+, CD3+, TCRb+, CD8a-, CD4+; Live CD3+ DN T cells as Zombie Aqualo, CD45+, CD3+, TCRb+, CD8a-, CD4-. (A) Representative flow cytometry dot plots for CD4 T cells (left panels) and CD8 T cells (right panels) for both the MAIT MR1 tetramer (MR1 5-OP-RU) and the MR1 negative control tetramer (MR1 6-FP). Gates, drawn to detect potential MAIT cells, are based off the negative control samples stained with MR1 6-FP. Numbers indicate percentage of cells within the drawn gates. (B) Representative flow cytometry dot plots for MR1 5-OP-RU and MR1 6-FP stained CD3+ DN T cells. [file Image2.tiff]
